# A *Drosophila* model of myeloproliferative neoplasm reveals a feed-forward loop in the JAK pathway mediated by p38 MAPK signalling

**DOI:** 10.1242/dmm.028118

**Published:** 2017-04-01

**Authors:** Ana Terriente-Félix, Lidia Pérez, Sarah J. Bray, Angel R. Nebreda, Marco Milán

**Affiliations:** 1 Institute for Research in Biomedicine (IRB Barcelona), The Barcelona Institute of Science and Technology, Baldiri Reixac, 10, 08028 Barcelona, Spain; 2 Department of Physiology, Development and Neuroscience, University of Cambridge, Cambridge CB2 3DY, UK; 3 ICREA, Pg. Lluís Companys 23, Barcelona 08010, Spain

**Keywords:** JAK, p38 MAPK, Myeloproliferative neoplasm, Haemocyte, Hypertrophy, *Drosophila*

## Abstract

Myeloproliferative neoplasms (MPNs) of the Philadelphia-negative class comprise polycythaemia vera, essential thrombocythaemia and primary myelofibrosis (PMF). They are associated with aberrant numbers of myeloid lineage cells in the blood, and in the case of overt PMF, with development of myelofibrosis in the bone marrow and failure to produce normal blood cells. These diseases are usually caused by gain-of-function mutations in the kinase JAK2. Here, we use *Drosophila* to investigate the consequences of activation of the JAK2 orthologue in haematopoiesis. We have identified maturing haemocytes in the lymph gland, the major haematopoietic organ in the fly, as the cell population susceptible to induce hypertrophy upon targeted overexpression of JAK. We show that JAK activates a feed-forward loop, including the cytokine-like ligand Upd3 and its receptor, Domeless, which are required to induce lymph gland hypertrophy. Moreover, we present evidence that p38 MAPK signalling plays a key role in this process by inducing expression of the ligand Upd3. Interestingly, we also show that forced activation of the p38 MAPK pathway in maturing haemocytes suffices to generate hypertrophic organs and the appearance of melanotic tumours. Our results illustrate a novel pro-tumourigenic crosstalk between the p38 MAPK pathway and JAK signalling in a *Drosophila* model of MPNs. Based on the shared molecular mechanisms underlying MPNs in flies and humans, the interplay between *Drosophila* JAK and p38 signalling pathways unravelled in this work might have translational relevance for human MPNs.

## Abstract (precis)

**Summary:** Pro-tumourigenic crosstalk occurs between the p38 MAPK pathway and JAK signalling in a *Drosophila* model of myeloproliferative neoplasm.

## INTRODUCTION

Myeloproliferative neoplasms (MPNs) arise in patients having a gain-of-function mutation in Janus kinase 2 (JAK2) or the myeloproliferative leukaemia protein receptor (MPL). Three specific subtypes of MPN occur, polycythaemia vera, essential thrombocythaemia or primary myelofibrosis (PMF), depending on the blood cell type whose concentrations are outside the homeostatic range. Although these subtypes are less severe than some other types of MPNs, such as chronic myelogenous leukaemia, which is caused by the translocation BCR-Abl (Philadelphia chromosome), 15% of patients exhibit PMF and a small percentage develop acute myeloid leukaemia, both of which compromise life expectancy. In 2005, *JAK2^V617F^* was identified as one of the most common mutations causing the disease (Baxter et al., 2005; James et al., 2005; Kralovics et al., 2005; Pecquet et al., 2010). Subsequently, this mutation was shown in murine models to be sufficient to induce activation of the JAK2 pathway in the bone marrow, and to increase the rates of proliferation of myeloid cells (Lacout et al., 2006). Long before the causal role of *JAK2^V617F^* in MPNs was known, *Drosophila* JAK gain-of-function mutations were shown to cause hypertrophy of the fly haematopoietic organs (lymph glands), and enhanced proliferation of circulating blood cells (haemocytes) and melanotic tumours (Corwin and Hanratty, 1976; Luo et al., 1997; Minakhina and Steward, 2006; Myllymäki and Rämet, 2014; Sorrentino et al., 2002).

In *Drosophila*, the conserved JAK/STAT signalling pathway is activated when ligands Unpaired (Upd) 1, 2 or 3, four-helix bundle cytokines of the Interleukin-6 family (Oldefest et al., 2013), bind to homodimers of the receptor Domeless (Dome), a type I cytokine receptor (Brown et al., 2001). This interaction promotes the anchoring of two JAK molecules at the intracellular domain of Dome, which allows JAK (also known as Hopscotch or Hop) trans-phosphorylation. Activated JAK then phosphorylates the transcription factor Stat92E, inducing its dimerization and nuclear translocation to promote transcription (Müller et al., 2005; Rivas et al., 2008). Functionally, JAK/Stat92E signalling is known to positively regulate cell proliferation. It does so in different cellular contexts under homeostatic conditions, and also, in response to stress signals. For instance, it is particularly important at sites of wound healing (Katsuyama et al., 2015; Santabárbara-Ruiz et al., 2015), in cells that lose their apico-basal polarity (Bunker et al., 2015) and in cells that experience chromosomal instability (Clemente-Ruiz et al., 2016), as well as regulating the growth of epithelial primordia (Mukherjee et al., 2005; Recasens-Alvarez et al., 2017). Similarly, the JAK pathway is required in the midgut epithelia for normal cell lineage differentiation and proliferation (Beebe et al., 2010), a requirement that is strongly evidenced under bacterial infection or stress assaults (Buchon et al., 2009; Cronin et al., 2009; Jiang et al., 2009). In the lymph gland, JAK signalling is required for the maintenance of progenitors in a naïve state (Gao et al., 2009), whereas peripheral tissues subjected to stress respond to the secretion of the ligand Upd3 by circulating haemocytes (Pastor-Pareja et al., 2008; Yang et al., 2015; Agaisse et al., 2003).

Another pathway that responds to stress is the p38 mitogen-activated protein kinase (p38 MAPK) cascade. In vertebrates, the p38 MAPK pathway can regulate cell cycle arrest, apoptosis or senescence, as well as the production of inflammatory mediators (Cuadrado and Nebreda, 2010). In *Drosophila*, the structurally and functionally conserved p38 MAPK signalling pathway is activated upon heat-shock (Inoue et al., 2001; Seisenbacher et al., 2011), osmotic stress (Inoue et al., 2001; Sano et al., 2005; Seong et al., 2011; Seisenbacher et al., 2011) and oxidative stress (Vrailas-Mortimer et al., 2011; Santabárbara-Ruiz et al., 2015; Clemente-Ruiz et al., 2016), and promotes survival upon exposure to chromosomal instability (Clemente-Ruiz et al., 2016), oxidative stress (Craig et al., 2004; Cai et al., 2011; Vrailas-Mortimer et al., 2011) and pathogenic bacteria (Chen et al., 2010; Ha et al., 2009; Park et al., 2009). The physiological role of the p38 MAPK signalling pathway in the lymph gland and its potential contribution to how these cells cope with stress conditions remain to be elucidated.

Here, we report a *Drosophila* model of MPNs based on forced expression of *JAK* (*hop*) in the lymph gland, and identify the maturing haemocytes as the cell population susceptible to induce JAK-induced hypertrophy. We unravel a feed-forward loop in the JAK/STAT pathway that involves the ligand Upd3 and its receptor Dome, and contributes to JAK-induced hypertrophy. We also show that the p38 MAPK pathway contributes to this feed-forward loop by regulating expression of the ligand Upd3, and, most interestingly, when activated in maturing haemocytes, suffices to induce lymph gland hypertrophy and melanotic tumours.

## RESULTS

### Targeted expression of JAK in maturing haemocytes induces lymph gland hypertrophy

**Fig. 1.**
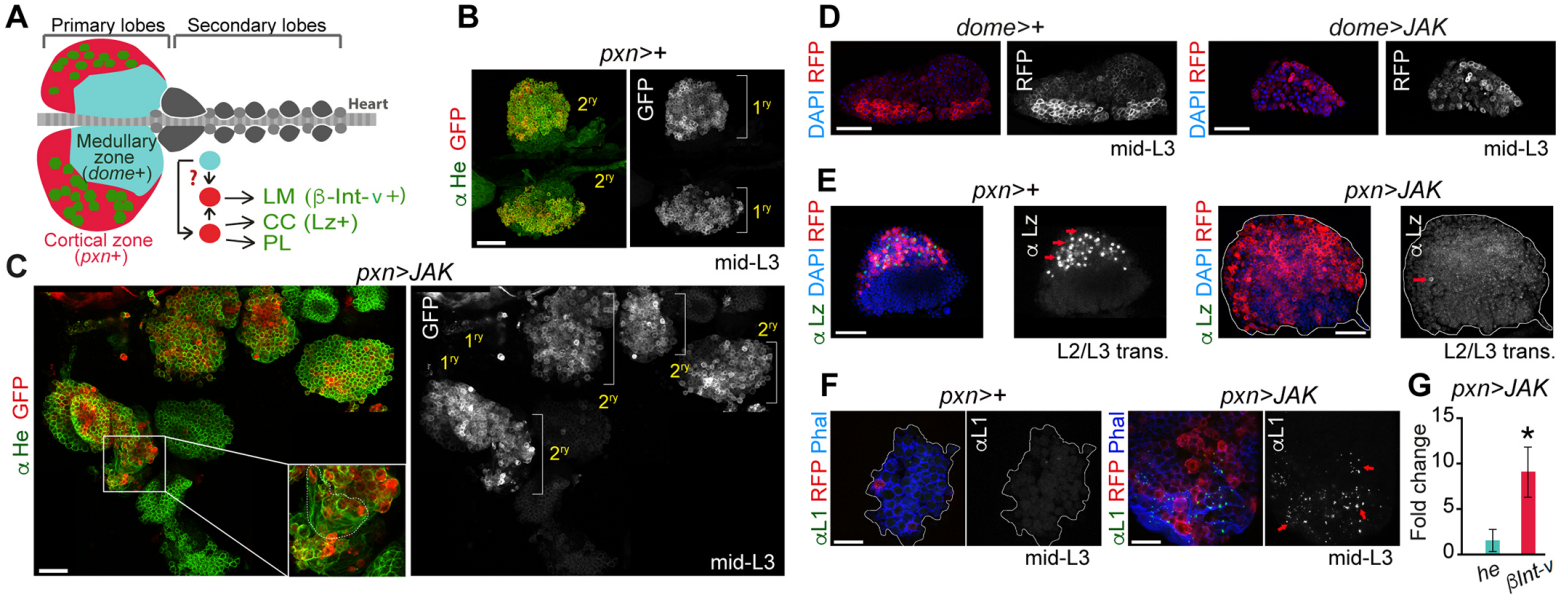
**Hypertrophic lymph glands induced by JAK overexpression in the cortical zone.** (A) Schematic of the primary and secondary lobes indicating the medullary zone (MZ, *dome*+) and cortical zone (CZ, *pxn*+), and the three different cell types: crystal cells (CC, Lz+), plasmatocytes (PL) and lamellocytes (LM, βInt-ν+). (B-F) Larval lymph glands of the indicated genotypes were labelled to visualise Hemese (He, green, B,C), RFP (red or white, B-F), DAPI (D,E), Lozenge (Lz, green or white, E) and Atilla/L1 (L1, green or white, F). CZ (*pxn-gal4*) or MZ (*dome-gal4*) drivers were used to express RFP and/or a wild-type form of JAK in the lymph gland of mid third-instar larvae (mid-L3; 91-94 h AEL, B,C,F) or larvae at the L2-L3 transition (E). Note in C that the secondary lobes grow in JAK-overexpressing lymph glands and the primary lobes have released their content. Inset in C shows a higher magnification of an overgrown secondary lobe consisting of large and elongate-shaped lamellocytes. Single images of a larger area have been assembled in C to show an overgrown lymph gland induced by JAK overexpression. Red arrows in E indicate Lz-positive cells. Red arrows in F highlight the presence of the lamellocyte marker L1. (G) mRNA levels of *βInt-ν* and *hemese* (*he*) measured as the mean±s.e.m. increase in JAK-overexpressing lymph glands compared with wild-type lymph glands. Expression of the *βInt-ν* lamellocyte-specific gene increases (fold change=9.1, *P*=0.039), whereas the expression of the haemocyte-specific gene *he* does not change significantly (fold change=1.55, *P*=0.31). Wild-type controls were given the value of 1 and are not displayed in the figure. *P<0.05. Scale bars: 40 µm (B-E), 20 µm (F).

Animals bearing the *JAK^Tum-l^* gain-of-function mutation, a hyperactive form of JAK, show hypertrophic lymph glands. This hypertrophy can also be obtained by targeted overexpression of a wild-type form of JAK to this organ (Harrison et al., 1995). The *Drosophila* larval lymph gland is composed of five to seven pairs of posterior secondary lobes and one pair of anterior primary lobes. Primary lobes are mainly subdivided into two domains: the medullary zone (MZ) and the cortical zone (CZ) (Jung et al., 2005). Naïve progenitors residing in the MZ progress into the CZ to differentiate (reviewed in Martinez-Agosto et al., 2007). In healthy larvae, progenitors residing in the CZ give rise to two cell types: the crystal cells (CCs, platelet-like cells) and the plasmatocytes (PLs, macrophage-like cells; Fig. 1A). In larvae parasitized by wasp eggs, progenitors differentiate into a third cell type, lamellocytes (LMs) (Jung et al., 2005). In order to identify the cell domain that is susceptible to over-proliferation upon JAK overexpression, a wild-type form of JAK was overexpressed in the MZ and CZ domains by the use of the *dome-Gal4* and *pxn-Gal4* drivers, respectively (Fig. 1A). The size of the resulting lymph glands and of the JAK-overexpressing domains was analysed in mid third-instar larvae [mid-L3; 91-94 h after egg laying (AEL)]. When JAK was overexpressed in the *pxn+* population, lymph glands were significantly larger than controls in this developmental stage (Fig. 1B,C). By contrast, expression of JAK in the *dome+* population resulted in fewer *dome*+ cells and smaller glands than controls (Fig. 1D). The overgrown glands in *pxn>JAK* primarily comprised enlarged secondary lobes, whereas primary lobes remained after apparent release of their cell contents (Fig. 1B,C, RFP, white channel, primary and secondary lobes). Such ‘bursting’ normally only occurs at metamorphosis and must be greatly accelerated in the *pxn>JAK* animals. In addition, the small number of *pxn*+ cells which are normally present at mid-L3 in wild-type glands (Fig. S1A, wild type, *pxn>+*) must become greatly expanded upon overexpression of JAK.

In order to identify the stage at which JAK induces growth of the *pxn*+ population in the primary lobes, we analysed the size of JAK-overexpressing lymph glands at early stages of larval development. We focused particularly on the transition between second- to third-instar larvae (L2-L3 transition; 69-72 h AEL) as this stage was previously shown to be critical for the generation of melanotic tumours in a *JAK^Tum-l^* background (Hanratty and Dearolf, 1993). We found that the *pxn-Gal4* driver started to be expressed in wild-type lymph glands 6 h prior to the L2-L3 transition (Fig. S1B, wild type, *pxn>+*). Interestingly, JAK-overexpressing glands showed a faster growth rate than controls across all time points analysed, which resulted in larger glands with a larger population of *pxn*+ cells (Fig. S1C, *pxn>JAK*). Furthermore, these primary lobes did not, at this stage, show signs of having burst and released their cell content to the haemolymph*.* Since each primary lobe could be analysed individually, we selected the developmental stage at the L2-L3 transition for further characterisation of the lymph gland hypertrophy caused by JAK overexpression (see below).

To investigate the similarities between the *JAK^Tum-l^* mutant and JAK overexpression, we analysed the cell differentiation state. Larvae mutant for *JAK^Tum-l^* showed melanotic tumours, which consist of aggregates of lamellocytes (Minakhina and Steward, 2006), and a reduced number of crystal cells in circulation (Hanratty and Dearolf, 1993; Harrison et al., 1995). When JAK was overexpressed in the *pxn*+ cell population, crystal cells, visualised by the expression of Lozenge (Lz+; Jung et al., 2005), rarely differentiated (Fig. 1E, red arrows). In these lymph glands, a multitude of large, elongated lamellocytes were detected (Fig. 1C, inset). These cells were also identified by expression of the specific lamellocyte marker Atilla/L1 (Kurucz et al., 2007) (Fig. 1F). Accordingly, in the *pxn>JAK* glands, we detected a significant increase in the expression levels of the lamellocyte-specific gene β-integrin-ν (*βInt-ν*; Kwon et al., 2008) compared with the pan-haemocyte marker *hemese* (*he*) (Jung et al., 2005) (Fig. 1G). Taken together, these results indicate firstly that *pxn+* cells are the most susceptible cell population to outgrow upon JAK overexpression, and secondly, that JAK induces a cell fate shift towards lamellocyte differentiation, at the expense of the crystal cells. Whether the increased number of lamellocytes observed in JAK-overexpressing lymph glands arises through the active proliferation of a normally quiescent lamelloblast population (Anderl et al., 2016) or through a programme of divisions and cell fate respecification amongst the plasmatocytes, remains to be elucidated.

### An Upd3-mediated feed-forward loop contributes to JAK-induced lymph gland hypertrophy

To analyse the physiological role of JAK/STAT in the *pxn+* cell population, we knocked down *JAK* expression using *JAK^RNAi^* and quantified the percentage of *pxn*+ cells in each lymph gland at mid-L3. The resulting primary lobes displayed no significant changes in the proportion of cells in the *pxn+* population when compared with wild-type controls (Fig. S2A). Similarly, when we expressed a truncated form of the receptor Dome, which lacks the intracellular domain (Dome^ΔCYT^; Brown et al., 2001), we did not observe any changes in the percentage of *pxn*+ cells per gland (Fig. S2A, *pxn>dome*^Δ*CYT*^). As control, we knocked down *JAK* in the *dome*+ cell population, and observed at mid-L3 a reduced number of *dome*+ cells in the MZ (Fig. S2B, MZ), which gave rise to smaller lymph glands (Fig. S2B, Total). This is consistent with the proposed role of JAK signalling in regulating the proliferation and/or survival of the cells residing in the MZ (Makki et al., 2010). Thus, our results indicate that the JAK/Dome pathway is either not required or has a redundant role with other signalling pathways during normal CZ development.

**Fig. 2.**
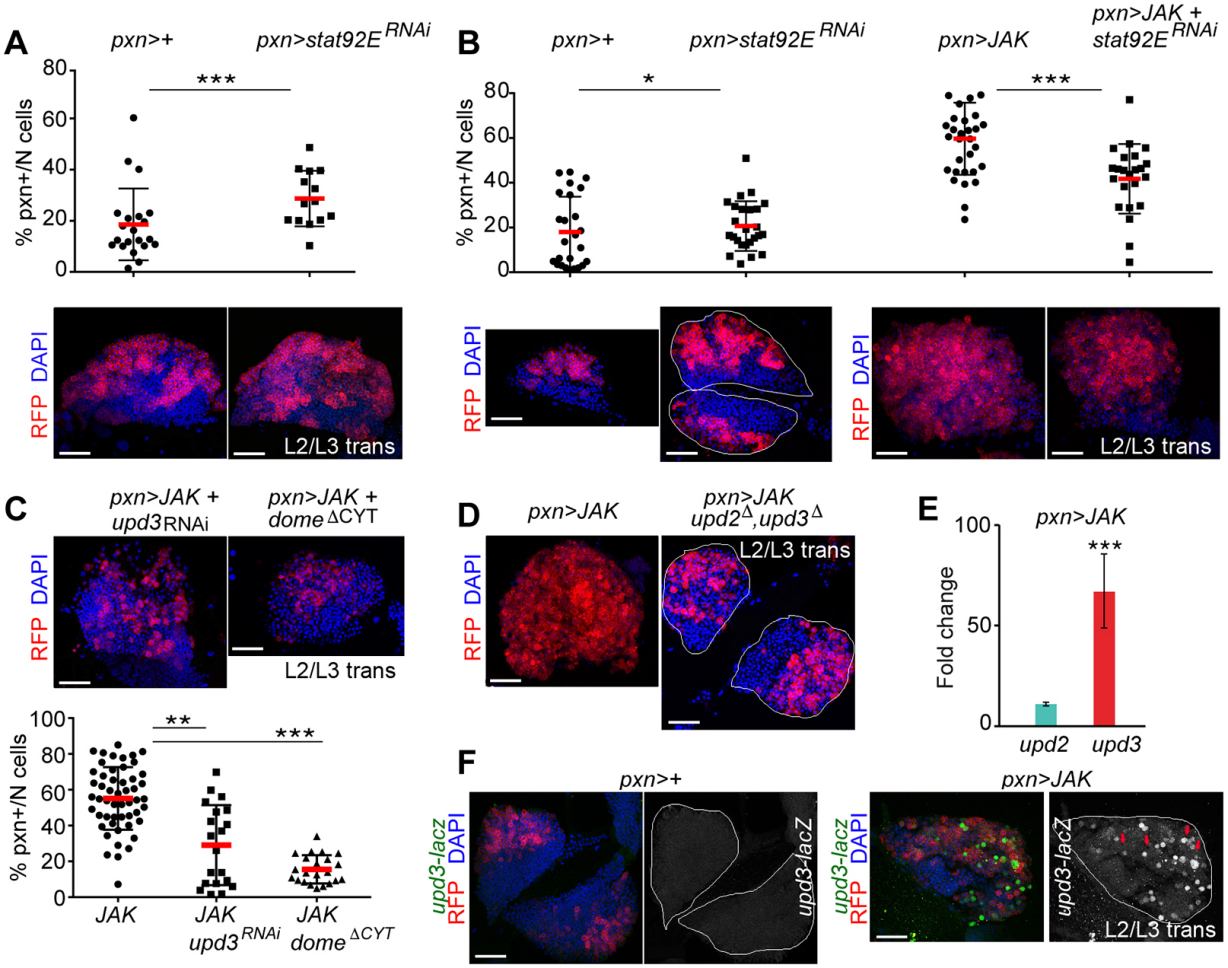
**JAK-induced hypertrophy requires Stat92E, Dome and Upd3.** (A-C) Scatterplots and immunofluorescence images showing the proportion of *pxn*+ cells per primary lobe (% *pxn*+/*N* cells) and lymph glands expressing the indicated transgenes under the control of the *pxn-Gal4* driver. Lymph glands were extracted from mid-L3 larvae (A) or larvae aged at the L2-L3 transition (B,C) and labelled to visualise RFP (red), and DAPI (blue). (A) Expansion of the *stat92E^RNAi^*-expressing cell population compared with the control *pxn>+* cell population (*P*=0.0087; *pxn>+*, *n*=21; *pxn>stat92E^RNAi^*, *n*=14). (B) Knockdown of *stat92E* partially rescues the JAK-induced expansion of the *pxn*+ population (*P*=0.0004; *pxn>JAK*, *n*=33; *pxn>JAK+stat92E^RNAi^*, *n*=23) whereas *stat92E^RNAi^* produces a subtle increase in the proportion of *pxn*+ cells compared with the control *pxn>+* cell population (*P*=0.03; *pxn>+*, *n*=25; *pxn>stat92E^RNAi^*, *n*=21). (C) Knockdown of *upd3* or expression of Dome^ΔCYT^ reduces the JAK-induced expansion of the *pxn*+ population (*pxn>JAK*+*dome^ΔCYT^*: *P*=1.961e^−10^, *n*=22; *pxn>JAK*+*upd3^RNAi^*: *P*=0.00234, *n*=20; *pxn>JAK*, *n*=44). (D) Lymph glands expressing JAK under the control of the *pxn-Gal4* driver were extracted and labelled as in B,C. Note the reduction in the expansion of the *pxn*+ cell population in *upd2^Δ^, upd3^Δ^* mutant lymph glands. (E) Increases in mRNA levels of *upd3* and *upd2* in lymph glands expressing JAK under the control of the *pxn-gal4* driver when compared with controls, which were given the value of 1. Note a significant increase in the expression level of *upd3* (fold-change=72.45, *P*=0.00013) but not in the expression level of *upd2* (fold-change=8.32, *P*=0.15). (F) Lymph glands expressing the indicated transgenes under the control of the *pxn-Gal4* driver were extracted from larvae aged at the L2-L3 transition and labelled to visualise *upd3-lacZ* expression (antibody against βGal, green or white), RFP (red), and DAPI (blue). Note induction of *upd3-lacZ* expression in *JAK*-overexpressing lymph glands. *P<0.05; **P<0.01; ***P<0.001. Every dot represents a single primary lobe. Red horizontal bar represents the mean, and whiskers represent 5% and 95% percentiles. The contour of the lymph glands is marked in B,D and F. Scale bars: 40 µm (A-D,F).

The function of the endogenous STAT (Stat92E) in the CZ was investigated by examining at mid-L3 the effect of expressing *stat92E^RNAi^* in the *pxn*+ cell population. As previously reported (Minakhina et al., 2011; Mondal et al., 2011), lymph glands with *stat92E* knockdown resembled, although to a milder extent, the phenotype resulting from upregulation of *JAK*. This is shown by the expansion of the *pxn*+ cell population (Fig. 2A). Previous work in *Drosophila* has identified a non-canonical mechanism by which the unphosphorylated form of Stat92E maintains HP1a localisation and heterochromatin stability (Shi et al., 2008). We thus wondered whether the ability of JAK to induce hypertrophy of the *pxn*+ cell population relied, at least in part, on the release of Stat92E from the heterochromatin. To avoid JAK-overexpressing glands bursting, primary lobes were examined at the L2-L3 transition. At this developmental time, the effect of knocking down Stat92E in *pxn*-expressing cells was milder (Fig. 2B, compare *pxn>stat92E^RNAi^* with *pxn>+*). However, we observed that the co-expression of *stat92E^RNAi^* together with JAK did not enhance the JAK-induced hypertrophy (Fig. 2B). By contrast, and consistent with a canonical role of Stat92E in mediating JAK activity, the downregulation of Stat92E resulted in a significant rescue of the JAK-induced expansion of the *pxn*+ cell population (Fig. 2B, compare *pxn>JAK+stat92E^RNAi^* with *pxn>JAK*). These results indicate that Stat92E is required downstream of JAK to sustain the growth of the CZ, independent of its non-canonical role in the repression of the CZ expansion in wild-type conditions.

We next studied the requirement for the receptor Dome in JAK-induced hypertrophy. We observed that co-expression of the truncated receptor Dome^ΔCYT^ greatly reduced the expansion of the *pxn+* cell population caused by JAK overexpression (Fig. 2C). Since the receptor Dome was apparently required for JAK-induced lymph gland hyperplasia, we investigated whether its ligands were also involved. Consistent with the requirement for Dome, JAK overexpression in larvae homozygous for a deficiency depleting the coding sequences of *upd2* and *upd3* resulted in a considerable reduction of the *pxn+* cell population compared with JAK overexpression alone (Fig. 2D). This suggests that JAK requires both the receptor and its ligands to induce lymph gland overgrowth. Then, we directly analysed the expression of the ligands in hypertrophic glands by RT-qPCR and found that *upd3* was strongly upregulated upon JAK overexpression, whereas *upd2* was increased to a lesser extent (Fig. 2E). Consistent with this result, we found that an *upd3* enhancer, previously shown to be activated in *Drosophila* neoplastic tumours (Bunker et al., 2015), was expressed in scattered *pxn*+ cells overexpressing JAK but not in the wild-type glands (Fig. 2F). Using an *upd3-RNAi* form, we confirmed that the overproliferation of JAK-overexpressing *pxn*+ cells requires Upd3 (Fig. 2C). Taken together, we conclude that JAK induces a feed-forward loop that triggers *upd3* expression, which contributes to JAK-induced hypertrophy of the lymph gland.

### A role for p38 MAPK signalling in maturing haemocytes

**Fig. 3.**
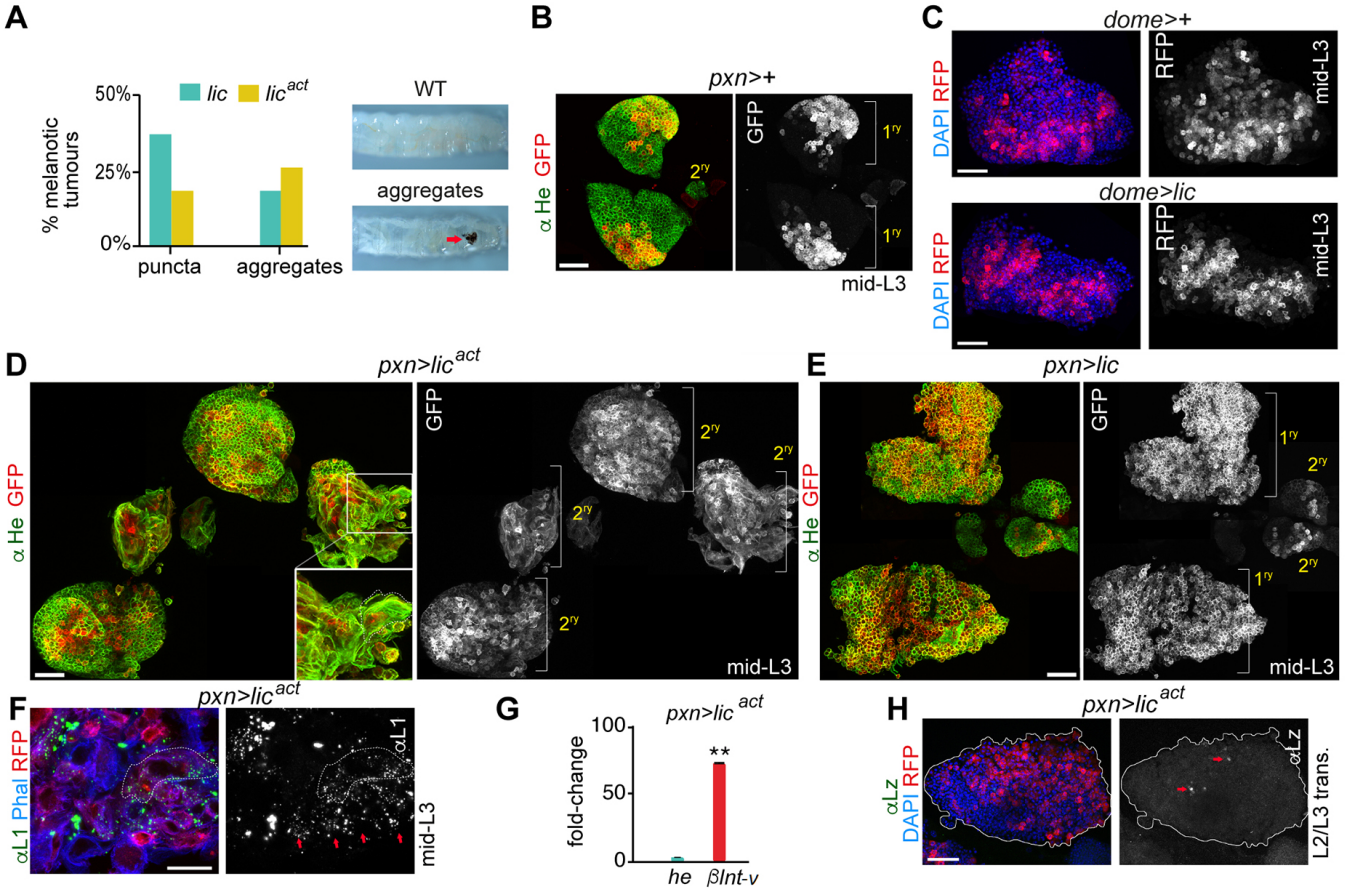
**Hypertrophic lymph glands induced by expression of an activated form of Licorne in the cortical zone.** (A) Histogram showing the percentage of larvae bearing small (puncta) or big (aggregates) melanotic tumours upon expression of wild-type (*lic*, green) or an activated form (*lic^act^*, yellow) of Licorne under the control of the *pxn-gal4* driver. Representative examples of a wild-type larva and of a larva bearing a big melanotic tumour are shown. *Lic*, *n*=93 larvae; *lic^act^*, *n*=109 larvae. (B,D,E) Lymph glands expressing the indicated transgenes under the control of the *pxn-Gal4* driver were extracted from mid third-instar larvae (mid-L3) and labelled to visualise RFP (red or white) and Hemese (He, green). Note that the secondary lobes grow in Lic^act^-expressing lymph glands (D), but their associated primary lobes have released their content. Inset in D shows a higher magnification of large, elongated lamellocytes. Also note that the primary lobes in Lic*-*overexpressing lymph glands (E) are larger than wild-type controls (B). (C) Lymph glands overexpressing Lic under the control of the *dome-Gal4* driver extracted from mid third-instar larvae (mid-L3) and labelled to visualise RFP (red) and DAPI (blue). (F) Expression of Lic^act^ in *pxn*+ cells induces differentiation of lamellocytes in mid-L3. Lamellocytes are distinguished by the expression of L1/Atilla (L1, green or white) and their large and elongated shape outlined by Phalloidin (Phal, blue); RFP visualises *pxn*+ cells (red). Note an increase of L1+ cells (red arrows). (G) Increased mRNA levels of *βInt-ν* and *hemese* (he) in lymph glands expressing Lic^act^ when compared with controls (fold-change increase of *βInt-ν*=76.50, *P*=0.03; fold-change of *he*=3.38, *P*=0.0052). Controls were given the value of 1 and are not displayed in the figure. **P<0.01. (H) Lymph gland expressing Lic^act^ under the control of the *pxn-Gal4* driver extracted from a larva at the L2-L3 transition and labelled to visualise RFP (red), Lozenge (Lz, green or white) and DAPI (blue). Note a reduced number of Lozenge-expressing cells (red arrows). The contour of the lymph gland is marked in H. Single images of a larger area have been assembled in D and E to show overgrown lymph glands induced by overexpression of Lic^act^ or Lic, respectively. Scale bars: 40 µm (B-E,H), 20 µm (F).

The p38 MAPK signalling pathway is an important regulator of cytokine and chemokine expression in mammals (reviewed by Clark and Dean, 2012; Cuadrado and Nebreda, 2010). We thus investigated the possible interplay between the p38 MAPK and JAK pathways in lymph gland hypertrophy. We first analysed the effect of expressing in the lymph gland a wild-type form of Licorne (Lic), the *Drosophila* protein kinase that activates p38a and p38b MAPKs (Adachi-Yamada et al., 1999; Han et al., 1998; Inoue et al., 2001), or an activated form (Lic^act^, see Materials and Methods for details). Interestingly, when Lic was overexpressed in the cortical zone with the *pxn-Gal4* driver, large melanotic aggregates were observed under the cuticle (Fig. 3A). The proportion of larvae bearing large melanotic aggregates was even higher upon overexpression of Lic^act^ in the *pxn*+ cell population (Fig. 3A). Whereas lymph glands overexpressing Lic showed larger primary lobes than controls (Fig. 3B,E, compare *pxn*>+ with pxn>lic), the overgrowth observed in *pxn>lic^act^* glands was due to the increase in size of secondary lobes as primary lobes showed signs of having released their cell content (Fig. 3D). At earlier stages of development, the primary lobes of *pxn>lic^act^* glands showed a similar growth pattern to the *JAK*-overexpressing glands (Fig. S1D). Moreover, Lic^act^-expressing glands showed numerous lamellocytes, as detected microscopically by their large size and elongated shape (Fig. 3D, left inset), by the expression of the lamellocyte marker Attila/L1 (Fig. 3F, red arrows, green and white channel) and by the elevated mRNA expression levels of the lamellocyte-specific marker *βInt-ν* (when compared with the pan-haemocyte marker *he*; Fig. 3G). In addition, lymph glands contained a reduced number of crystal cells labelled by the expression of Lozenge (Fig. 3H, red arrows, compare with Fig. 1E). Overexpression of Lic in the MZ produced primary lobes of about the same size as in the wild-type glands (Fig. 3C), whereas expression of Lic^act^ in the MZ caused larval lethality, most probably due to the expression of the *dome-Gal4* driver in the embryo. Altogether, these results indicate that activation of the Licorne/p38 MAPK signalling pathway in the *pxn*+ cell population phenocopies the effects of JAK overexpression and induces lymph gland dysplasia.

**Fig. 4.**
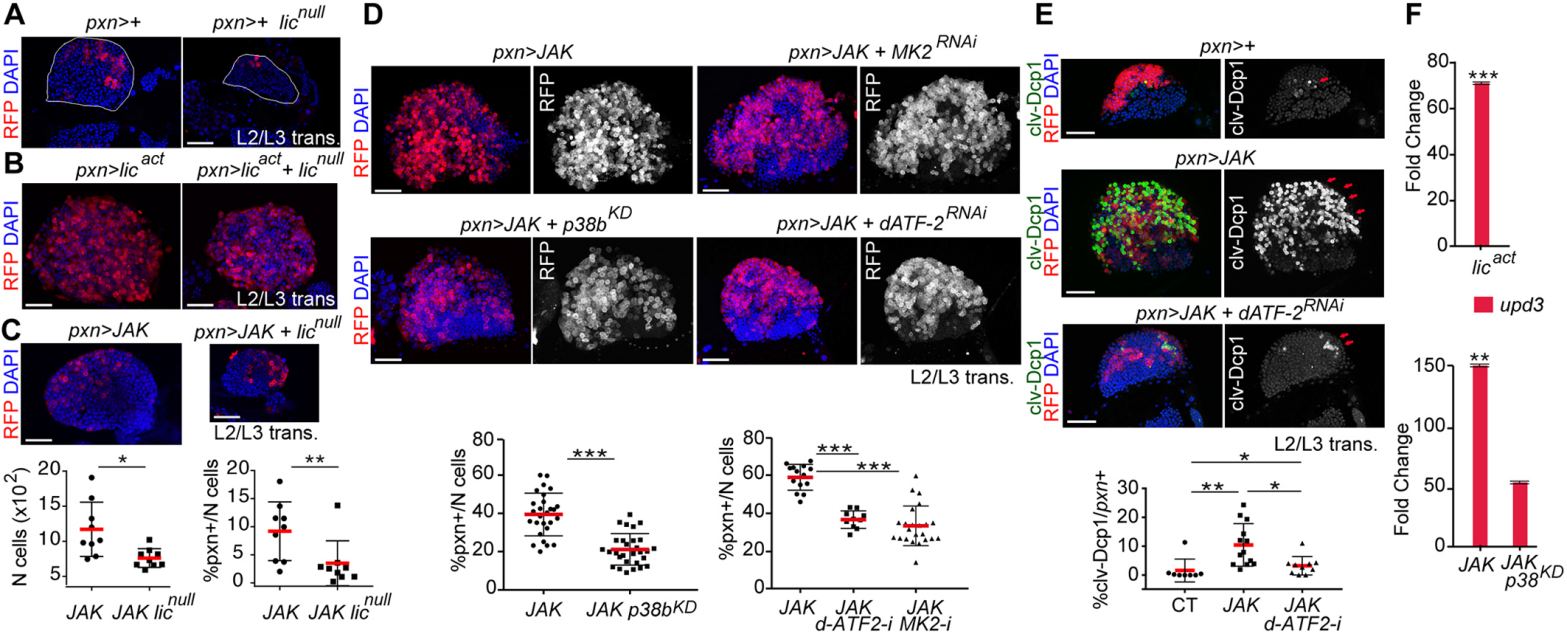
**A role for p38 MAPK signalling in JAK-induced hypertrophy.** (A) Expression of *pxn-Gal4* marked by the expression of RFP (red) in wild-type and in *lic^null^* hemizygous lymph glands (outlined). (B,C) Expression of Lic^act^ (B) or JAK (C) under the control of the *pxn-Gal4* driver in control and in *lic^null^* hemizygous lymph glands. Scatterplot in C shows the proportion of *pxn*+ cells per primary lobe (% *pxn*+/*N* cells), and the total cell number per primary lobe (*N* cells). Note that loss of *lic* induced a reduction in the number of *pxn*+ cells per primary lobe caused by JAK overexpression (*P*=0.006) and a reduction in the number of cells per lymph gland (*P*=0.034; *pxn>JAK*, *n*=9; *pxn>JAK+lic^null^*, *n*=9). (D) Genetic interactions between *p38b*, *dATF-2* and *MK2* and JAK in the *pxn+* cells. Scatterplots show the proportion of *pxn*+ cells per primary lobe (% *pxn*+/*N* cells) and immunofluorescence images show lymph glands expressing the indicated transgenes under the control of the *pxn-Gal4* driver. Note that expression of *p38b^KD^*, *MK2^RNAi^* or *dATF-2^RNAi^* reduced the number of *pxn*+ cells per primary lobe caused by JAK overexpression (*pxn>*JAK vs *pxn>JAK*+*p38b^KD^*, *P*=6.318e^−06^; *pxn>*JAK vs *pxn>JAK*+*dATF-2^RNAi^*, *P*=1.02e^−06^; *pxn>*JAK vs *pxn>JAK*+*MK2^RNAi^*, *P*=3.848e^−06^; *pxn>*JAK, left plot, *n*=27 and right plot, *n*=14; *pxn>JAK*+*p38b^KD^*, *n*=29; *pxn>JAK*+*dATF-2^RNAi^*, *n*=10; *pxn>JAK*+*MK2^RNAi^*, *n*=23). (E) Expression of the cleaved form of the effector caspase Dcp1 (clv-Dcp1, marked in green and white) increases upon JAK overexpression in *pxn*+ cells (*pxn>JAK* vs *pxn>+*, *P*=0.038; *pxn>*+, *n*=8; *pxn>JAK*, *n*=13) and was rescued by the expression of *dATF-2^RNAi^* (*pxn>JAK* vs *pxn>JAK+dATF-2^RNAi^*, *P*=0.001; *pxn>JAK*+*dATF-2^RNAi^*, *n*=12), although not to the levels observed in the wild type (*pxn>JAK+d-ATF2^RNAi^* vs *pxn>+*, *P*=0.035). (F) Increased mRNA levels of *upd3* in lymph glands expressing Lic^act^ (fold-change=71.1, *P*=0.0006), *JAK* (fold-change=158.43, *P*=0.0099) or *JAK* and *p38b^KD^* (fold-change=54.79, *P*=0.063) under the control of the *pxn-Gal4* driver when compared with controls (*pxn>+*), which were given the value of 1. In A-E, lymph glands of the different genotypes were extracted from larvae at the L2-L3 transition and labelled to visualise RFP (red or white) and DAPI (blue). *P<0.05; **P<0.01; ***P<0.001. In the scatterplots, every dot represents a single primary lobe; red horizontal bars represent the mean, and whiskers represent 5% and 95% percentiles. Scale bars: 40 µm.

To analyse whether the p38 MAPK pathway has a role in the lymph gland during normal development, we studied hemizygous *lic^null^* mutants. As previously described (Cully et al., 2010), larvae with reduced levels of Lic activity were smaller than wild-type larvae (data not shown). Consistently, their lymph glands were also smaller than lymph glands from wild-type larvae of the same developmental age (Fig. 4A). We next analysed the role of endogenous p38 MAPK signalling in the different regions of the lymph gland. For this purpose, we used RNAis targeting *p38a* and *p38b*, *lic* or the downstream transcription factor *dATF-2* (Han et al., 1998; Sano et al. 2005). Targeted expression of these RNAis to the CZ resulted in a reduced number of *pxn*+ cells compared with wild-type glands at mid-L3 (Fig. S3A). By contrast, targeted depletion of *lic* in the MZ did not reduce the number of *dome*+ progenitors and the lymph glands showed a subtle enlargement (Fig. S3B, Total). Consistent with a specific requirement of the Licorne/p38 MAPK signalling pathway in the CZ, expression of Lic^act^ under control of the *pxn-Gal4* driver produced lymph glands of similar size in both *lic^null^* mutant and wild-type control animals raised in parallel and visualised at the transition between L2 and L3 (Fig. 4B). Taken together, these results indicate that the p38 MAPK pathway has a role in regulating growth of the *pxn+* cell population during normal development.

### A role for p38 MAPK signalling in JAK-induced hypertrophy of the lymph gland

The above experiments indicate that activation of p38 MAPK signalling in the CZ phenocopies the JAK-induced lymph gland hypertrophy and the cell fate shift towards lamellocyte differentiation. In order to test whether p38 MAPK signalling contributes to the JAK-induced phenotype, we analysed the ability of JAK overexpression to induce hypertrophic lymph glands in a *lic^null^* mutant background. Interestingly, *lic*-deficient lymph glands showed a reduced expansion of the *pxn*+ cell population and a smaller size upon overexpression of JAK when compared with wild-type glands (Fig. 4C, compare *pxn>JAK* with *lic^null^; pxn>JAK*). Consistent with this result, co-expression of the kinase-dead form of p38b (*p38b^KD^*; which can act as a dominant-negative form blocking p38 MAPK signalling) with RNAi specific for MK2 [*MK2^RNAi^*; a protein kinase activated by p38 MAPK (Cuadrado and Nebreda, 2010)] or the transcription factor dATF-2 (*dATF-2^RNAi^*), were able to rescue the expansion of the *pxn*+ cell population caused by JAK overexpression (Fig. 4D). These data confirm that p38 MAPK signalling is required downstream of the JAK/STAT pathway to promote the expansion of the *pxn*+ cell population. We also observed that JAK overexpression induced high levels of apoptosis, as monitored by an antibody that detects the cleaved form of the effector caspase Dcp1, which was rescued by dATF-2 depletion (Fig. 4E). Whether the induction of cell death is a direct consequence of p38 MAPK activation or an indirect consequence of the enhanced proliferative capacity of the tissue upon JAK overexpression remains to be elucidated.

Next, we analysed whether p38 MAPK signalling is required downstream of the JAK pathway to regulate *upd3* expression. We found that lymph glands co-expressing JAK together with p38b^KD^ showed lower expression levels of *upd3* than lymph glands overexpressing JAK alone (Fig. 4F). In addition, expression of constitutively active Lic^act^ sufficed to upregulate *upd3* expression in lymph glands (Fig. 4F), and *upd3* was upregulated by Lic^act^ to a greater extent than ligands such as Spatzle or Eiger (Fig. S4A), which can activate the Toll and JNK pathways, respectively, and induce melanotic tumours (Qiu et al., 1998; Zettervall et al., 2004). We confirmed in Kc167 cells that expression of Lic^act^ sufficed to induce upregulation of *upd2* and *upd3* (Fig. S4B), and this required p38 MAPK activation because levels were reduced when Lic^act^ was expressed in the presence of the p38 MAPK inhibitor SB203580 (Fig. S4B). In order to test whether the increased expression of *upd2* and *upd3* resulted in activation of the JAK/STAT pathway, we analysed the activity of *6x2xDrafLuc*, a reporter widely used to measure the activity of this pathway (Thomas et al., 2015; Müller et al., 2005). Luciferase assays revealed increased reporter activity in Kc167 cells expressing Lic^act^ or JAK^Tum-l^, and to a lesser extent upon overexpression of wild-type JAK (Fig. S4C). Altogether, these results support the implication of the p38 MAPK signalling pathway in JAK-induced lymph gland hypertrophy by regulating *upd3* expression.

## DISCUSSION

Here, we have analysed the impact of JAK overexpression in the different cell populations of the lymph gland, the major haematopoietic organ of *Drosophila*. This has allowed us to identify the maturing haemocytes as the cell population that is responsible for JAK-induced hypertrophy. In addition, JAK-overexpressing lymph glands showed increased numbers of differentiated lamellocytes and fewer crystal cells, a phenotype that resembles the effect of *JAK^Tum-l^* mutants. Using this model, we have identified a number of essential components in the JAK/STAT pathway that have an important role in JAK-induced lymph gland dysplasia. First, the transcription factor Stat92E was found to be a necessary element downstream of JAK, independent of its role in preventing expansion of the cortical zone in wild-type conditions. This result concurs with previous publications showing that phosphorylation of Stat92E in *JAK^Tum-l^* haemocytes is required for the formation of melanotic tumours (Bausek and Zeidler, 2014; Remillieux-Leschelle et al., 2002; Sorrentino et al., 2004). We speculate that Stat92E regulates a set of genes in wild-type conditions that prevent the expansion of the *pxn+* population, and that these genes are different from those regulated upon JAK phosphorylation, which might be involved in dysplastic growth. This is consistent with a report showing that the unphosphorylated and phosphorylated forms of Stat5, the vertebrate orthologue of Stat92E, can regulate different sets of genes (Park et al., 2015). Most interestingly, we unravelled a requirement for the Dome receptor in JAK-induced hypertrophy, and identified the ligand Upd3 as an essential component involved in a feed-forward loop downstream of JAK signalling that contributes to lymph gland dysplasia. This work complements the use of phenotypic screenings based on the presence of melanotic tumours (Shi et al., 2008) and forward RNAi screenings (Müller et al., 2005) – genetic approaches that in the past served to identify new elements of the JAK regulatory network in haemocytes. Furthermore, our experimental setup could be useful to perform small-scale drug screenings or to validate hits previously identified in drug screenings performed in *Drosophila* cultured cells (Thomas et al., 2015). Of particular interest would be drugs that synergise with JAK inhibitors, since Ruxolitinib, an FDA-approved JAK inhibitor, does not ameliorate MPN symptoms in the long term (Mascarenhas et al., 2014).

Our genetic model of MPNs has also allowed us to identify a novel role for the p38 MAPK pathway in the feed-forward loop downstream of JAK signalling that contributes to lymph gland dysplasia. The p38 MAPK pathway exerts is function by regulating *upd3* expression. Importantly, we found that overexpression of Licorne, a direct and specific activator of p38 MAPKs, suffices to induce dysplasia and phenocopies the effect of JAK overexpression. Furthermore, p38 MAPK signalling was able to induce *upd3* expression in lymph gland cells and in Kc167 cells. Our data show that the p38 MAPK pathway, including the transcription factor dATF-2, is necessary for the maturing haemocytes to proliferate both in wild-type conditions and in response to *JAK* overexpression. Similarly, there is evidence that p38 MAPK signalling can stimulate mammalian cell proliferation in particular contexts, for example, in mouse colon tumour cells (Gupta et al., 2014). Megakaryocyte proliferation induced by FLT3 receptor activation has also been reported to involve p38 MAPK signalling (Desterke et al., 2011). In addition, the p38 MAPK pathway plays an important role in the regulation of cytokine expression both at transcriptional and post-transcriptional levels (reviewed by Cuadrado and Nebreda, 2010; Tiedje et al., 2014). However, we are not aware of any report showing that direct activation of the p38 MAPK pathway by an upstream regulator such as Lic suffices to induce dysplasia and tumour formation *in vivo*. On the contrary, p38 MAPK hyperactivation usually leads to cell cycle arrest and cell death in mammalian cells (Cuadrado and Nebreda, 2010; Tiedje et al., 2014). This suggests that haemocytes, and perhaps the concurrent activation of JAK signalling, may provide a particular context that favours a pro-tumourigenic role for p38 MAPK signalling. Intriguingly, there are no reports on mutations, changes in copy number, promoter methylation or enhanced phosphorylation levels of p38 MAPKs in samples from MPN patients (Desterke et al., 2011; Shahjahan et al., 2008), supporting our conclusion that p38 MAPK signalling contributes to tumourigenesis as part of the JAK-triggered feed-forward loop. Given that MPN patients are known to have high levels of circulating cytokines (Levine et al., 2007; Tyner et al., 2010), it is tempting to speculate that p38 MAPK signalling contributes to *JAK2^V617F^*-associated mammalian tumourigenesis by regulating the production of cytokines, which act in an autocrine manner.

## MATERIALS AND METHODS

### Fly strains

The alleles and fly stocks, as described in FlyBase (flybase.org/), were *pxn-Gal4* (Stramer et al., 2005), *dome-Gal4* [#PG125 (Makki et al., 2010)], *UAS-cd8RFP* (BDSC #32219 and #32218), *UAS-cd8GFP* (BDSC #5030), *upd3-lacZ* (Bunker et al., 2015), *UAS-JAK^HA^* (FlyORF #F001803), *UAS-lic* (FlyORF #F001674), *UAS-stat92E^RNAi^* (VDRC #106980), *UAS-upd3^RNAi^* (VDRC #27134), *UAS-dome^ΔCYT^* (Brown et al., 2001), *UAS-p38b^KD^* (Vrailas-Mortimer et al., 2011), w*,upd2^Δ^,upd3^Δ^ (BDSC #55729), *lic^D13^* [*lic^null^* in the text (Cully et al., 2010)], *UAS-JAK^RNAi^* (BDSC #32966), *UAS-p38a^RNAi^* (VDRC #52277), *UAS-p38b^RNAi^* (VDRC #108099), *UAS-lic^RNAi^* (VDRC #106822), *UAS-dATF-2^RNAi^* (DGGR #3749-R2 and BDSC #60124) and *UAS-MK2^RNAi^* (VDRC #3170).

*UAS-lic^RNAi^ and *UAS-stat92E^RNAi^* were VDRC lines of the KK collection. Because of the presence of a landing site in the gene *tiptop*, lines were meiotically recombined to acquire the dominant mutation Sco. Then, flies were PCR screened for the absence of *tiptop* landing site and the presence of the correct non-annotated landing site at cytological position 40D. Then the Sco mutation was removed by meiotically recombining the arm with a wild-type chromosome. Full genotypes of flies used for results displayed in all figures are listed in supplementary Materials and Methods.

### Generation of the UAS-*lic^act^* construct

A *lic* ORF frame (SD04985; 321-1325 bp) was used to make a phospho-mimetic version, by swapping serine (S200) and threonine (T204) residues to aspartate (D). The mutated *lic* was cloned into *pUAS* to transform *yw* flies (Brand and Perrimon, 1993).

### Generation of cell lines

Stable lines of Kc167 cells were prepared and treated as described in supplementary Materials and Methods.

### RT-qPCR

Real-time quantitative PCR was carried out on total RNA from lymph glands using primers listed in supplementary Materials and Methods.

### Experimental setup

For Figs 1-4, Figs S2 and S3, lymph glands were extracted from larvae that were either 69-72 h AEL (L2-L3 transition) or at 91-94 h AEL (mid-L3). To age the larvae, eggs were collected every 3 h at 25°C and shifted to 29°C at 45-48 h AEL. In this manner, incubating lymph glands at 29°C for 24 h or 48 h, we increased the efficiency of the Gal4/UAS system. For Fig. S1B-D, eggs were collected every 3 h. Larvae were cultured at 25°C until 63-66 h AEL (6 h before the L2-L3 transition). Then they were shifted to 29°C and lymph glands were dissected 6 h before the L2-L3 transition, at the L2-L3 transition and 6 h later.

### Antibodies and dyes

Antibodies used were: anti-He [1:30, mouse; Istvan Ando, Institute of Genetics Hungarian Academy of Sciences, Hungary (Kurucz et al., 2003)]; anti-Atilla/L1a,b,c [1:10, mouse; Istvan Ando (Kurucz et al., 2007)]; anti-Lz (1:100, mouse, concentrated; DSHB); anti-βGal (1:1000, mouse; DSHB, 40.1a); anti-GFP (1:300, goat; Abcam, ab6673); anti-cleaved-Dcp1 (clv-Dcp1; 1:100, rabbit; Cell Signaling Technology, 9578); Alexa Fluor 488 Phalloidin (1:50; Cell Signaling Technology, 8878). Secondary antibodies for IF were from Life Technologies and Jackson Immunoresearch.

### Immunofluorescence and imaging

Ten lymph gland pairs were settled on poly-L-lysine-coated slides with a silicon well containing PBS. Samples were fixed for 20 min with 4% paraformaldehyde at room temperature (RT), rinsed three times with 1×PBS, 5 min with 1×PBS+1% Triton X-100 (PBST) and then incubated for 1 h with PBST plus 4% horse serum at RT. Next, samples were incubated overnight at 4°C with primary antibodies. The following day, samples were rinsed and washed three times for 15 min with PBST, incubated for 1 h and 15 min with secondary antibodies, washed three times for 15 min with PBST and once with 1×PBS. To finish the mounting, the silicon well was removed and glycerol-based medium containing DAPI (2 mg/ml) was added. Confocal images were taken using a confocal Leica TCS SP5 microscope.

For bright-field images, live larvae were held in a drop of iced water and photographed under an Olympus MVX10 microscope.

### Cell imaging and processing

Cell imaging and processing were performed using Fiji 2.0.0, MATLAB R2016a and Adobe Photoshop CC 2015. The brightness of all figure panels was adjusted to normalize RFP expression levels, which labels the transgene-expressing population and can vary from sample to sample.

### Cell quantification in primary lobes

To quantify the total number of cells per primary lobe (N cells), individual cells were considered as nuclei detected by the maximal local intensity in the DAPI channel. The nuclei were detected in two to three confocal planes. Original data were filtered using a Laplacian filter. To measure the proportion of cells that expressed *pxn>mRFP* in a single primary lobe (% *pxn*+/*N* cells), we detected the channel RFP in shells whose centre was the maximal detecting point of the DAPI. This shell was designed to have an empty gap of the nuclei diameter in order to capture the intensity values showed by cytoplasmic mRFP. Defining a threshold, we could discriminate as positive *pxn*+ cells those having 75% of the maximal local intensity. Measurements of *pxn+*/*N* cells described in the paper are linked to the ‘*N* cells’ measurement presented in Fig. S5.

### Statistical analyses

Between 10 and 35 samples (primary lobes) were used for each of the quantifications. Control and experimental samples were collected and analysed in parallel. We did not use any method for randomisation, blinding, exclusion of any sample or to measure the variances. *P-*values were calculated using Kolmogorov-Smirnov for nonparametric data. Analyses were done with Prism 7.0 (GraphPad) and R.
